# Supplementary material for: Imidazole-Based pH-Sensitive Convertible Liposomes for Anticancer Drug Delivery
Source: Pharmaceuticals (Basel). 2022 Mar 3;15(3):306. doi: 10.3390/ph15030306 (PMC8949415; doi:10.3390/ph15030306)
Supplement: Supplementary file 1 [file pharmaceuticals-15-00306-s001.zip › pharmaceuticals-1610343-supplementary.pdf]

SUPPLEMENTAL MATERIAL

Mass and NMR Spectra of Imidazole-based Lipids

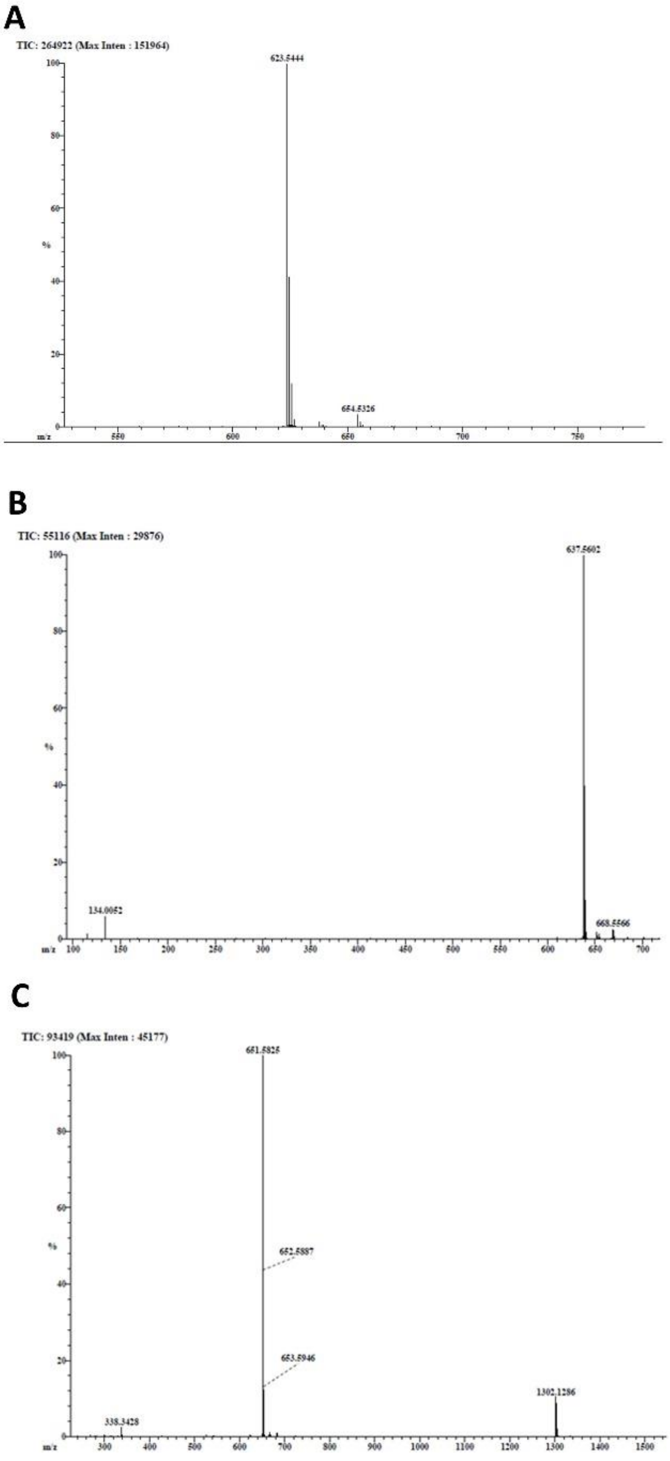

Figure S1. DART Mass Spectra of imidazole-based lipids DHI (A), DHMI (B), and DHDMI (C).

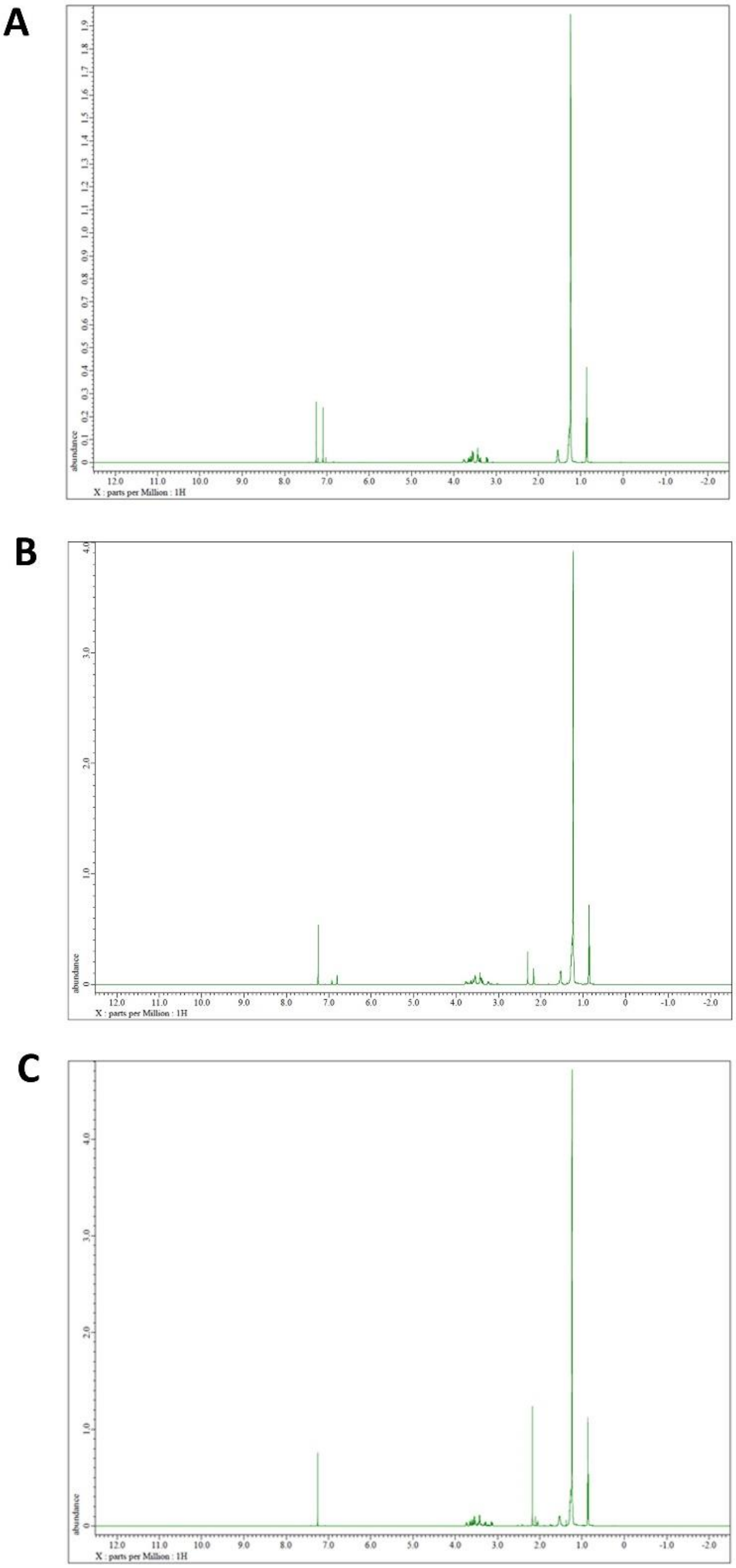

Figure S2. 1H-NMR Spectra (600 MHz, $CDCl_3$) of imidazole-based lipids DHI (A), DHMI (B), and DHDMI (C).

Growth and Morphology of 3D MCS

Cell Line	HeLa	A549	MDA-MB-231	MDA-MB-468
Seeding Density (cells/well)	500	5000	3000	2000
Collagen Concentration (%)	0	0.3	1	1
Centrifuge Speed (×g)	1000	300	200	200
Centrifuge Time (min)	15	7	15	15
Approximate Time to Reach 500 μm in Diameter (days)	12	5	5	11

Table S1. Conditions to culture 3D MCS of cancer cells.

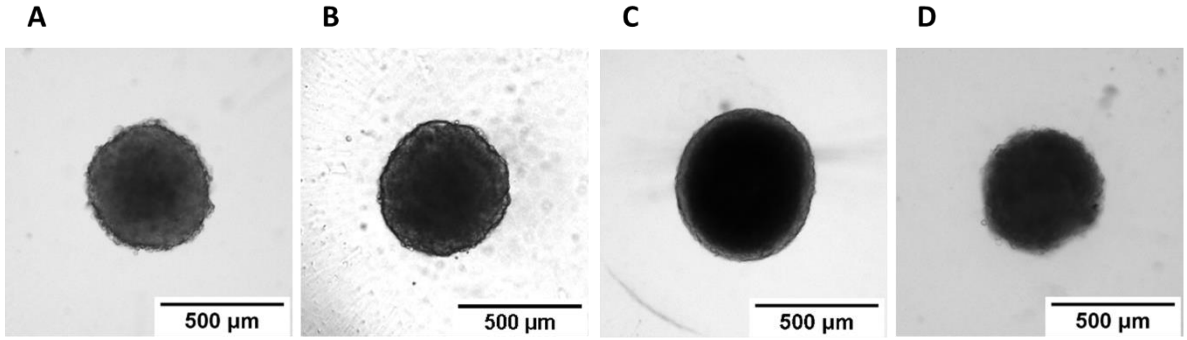

Figure S3. Representative morphology of MCS with diameter of ~500 μm in the ULA 96-well microplates for anticancer drug treatment: A, HeLa MCS (500 cells/well, no collagen, 12 days); B, A549 MCS (5000 cells/well, 0.3% collagen, 5 days); C, MDA-MB-231 MCS (3000 cells/well, 1% collagen, 5 days); D, MDA-MB-468 MCS (2000 cells/well, 1% collagen, 11 days).

pH Gradient in 3D MCS

Carboxy SNARF-1 was dissolved in anhydrous DMSO at concentration of 100 µM and added to 3D MCS in a glass bottom dish to a final concentration of 10 µM. The 3D MCS were then incubated for 45 mins at 37 °C, washed 3 times with PBS and imaged using a Leica DMIRE2 confocal microscopy. The 3D MCS loaded with Carboxy SNARF-1 was then exposed to three calibration solutions of 10 µM nigericin at pH 5.5, 6.8 and 8.0 as reported before [1]. Three calibration images at each pH were recorded as triplicates. Images were acquired using the MetaMorph software and analyzed using the ImageJ software. One z-stack picture at 200 µm above the bottom of each 3D MCS was selected for the analyses and divided into five areas using concentric circles. The mean value of the two emission fluorescence signals of each region was measured. The R value of each region was then calculated after subtracting the background noise. The corresponding pH value was then calculated by the equation:

$$\mathrm{pH} = \mathrm{p}K_a - \log[\frac{R-R_B}{R_A-R} \times \frac{F_{B(\lambda 2)}}{F_{A(\lambda 2)}}],$$

where the pKa of carboxy SNARF-1 is ~ 7.5, R is the ratio of the fluorescence intensities at the two wavelengths λ_1= 580 nm and λ_2= 640 nm ($F_{\lambda 1}/F_{\lambda 2}$). Subscripts A and B represent the limiting values at the acidic and basic endpoints, respectively.

According to the above equation, R value is inversely proportional to the pH value. The descending trend of R was observed from the central area to the peripheral area of the concentric circles (Area 1 to Area 5 in **Figure S4**). At the limiting acidic and basic conditions, R_A, R_B, $F_{A(\lambda 2)}$ and $F_{B(\lambda 2)}$ were selected from the area closer to the edge of the same z-stack, which was equilibrated to the calibrated medium pHs by the K+/H+ ionophore nigericin. The gradual increase of R from Area 5 to Area 1 indicated the decrease of the intra-MCS pH from the edge to the core (**Table S2**).

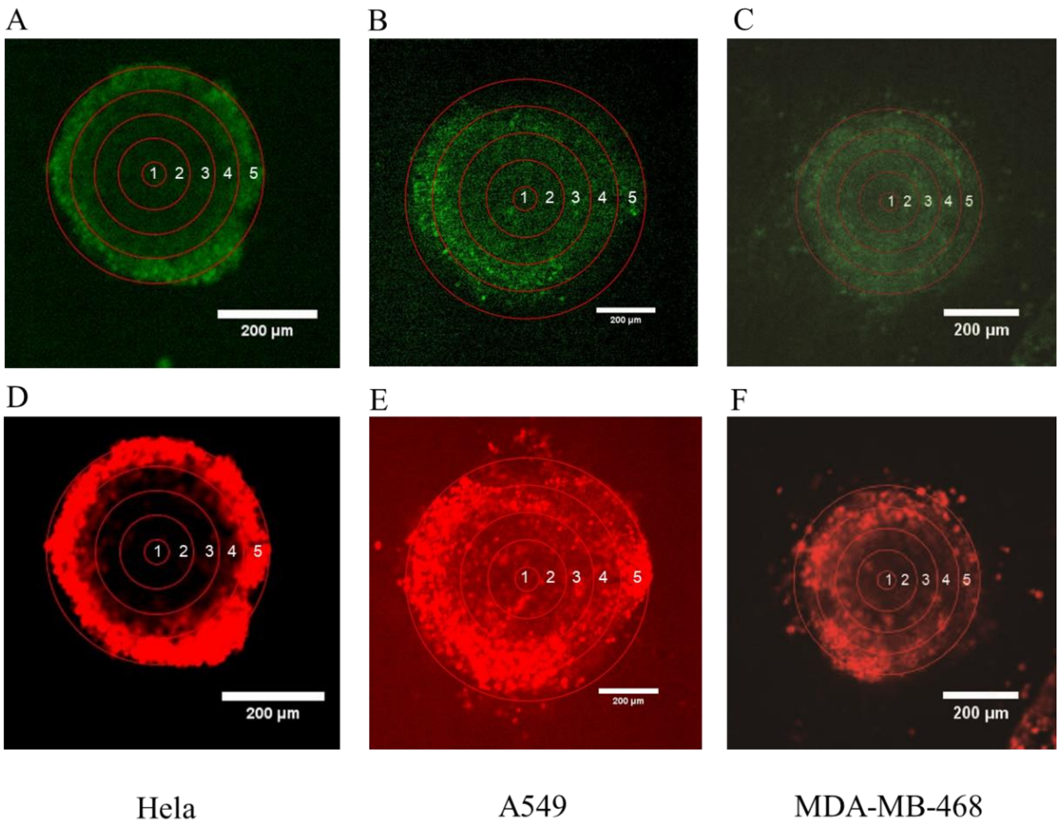

Figure S4. Representative confocal images of 3D MCS for intra-MCS pH measurements. The 3D MCS of Hela (A, D), A549 (B, E), and MDA-MB-468 (C, F) cells were incubated with Carboxy SNARF-1 and imaged at two channels: 580 nm/green (A, B, C) and 640 nm/red (D, E, F).

Area Number	HeLa	A549	MDA-MB-468
1	5.71	6.18	6.36
2	6.55	6.76	6.46
3	7.01	7.09	7.28
4	7.68	7.24	8.03
5	7.79	7.07	7.71

Table S2. Calculated pH in 3D MCS of Hela, A549, and MDA-MB-468 cancer cells based on fluorescent images of confocal microscopy. The areas are marked by concentric circles as shown in **Figure S4**.

References

1. Sun, B., C.H. Leem, and R.D. Vaughan-Jones, *Novel chloride-dependent acid loader in the guinea-pig ventricular myocyte: part of a dual acid-loading mechanism.* J Physiol, 1996. **495 (Pt 1)**: p. 65-82.
